# Evaluating the evidence for macrophage presence in skeletal muscle and its relation to insulin resistance in obese mice and humans: a systematic review protocol

**DOI:** 10.1186/s13104-017-2686-6

**Published:** 2017-08-08

**Authors:** Meha Bhatt, Srikesh Rudrapatna, Laura Banfield, Rachel Bierbrier, Pei-Wen Wang, Kuan-Wen Wang, Lehana Thabane, M. Constantine Samaan

**Affiliations:** 1 Department of Health Research Methods, Evidence, and Impact, McMaster University, Hamilton, ON Canada; 2 Department of Pediatrics, McMaster University, Hamilton, ON Canada; 3 Medical Sciences Graduate Program, McMaster University, Hamilton, ON Canada; 4 Division of Pediatric Endocrinology, McMaster Children’s Hospital, Hamilton, ON Canada; 5 Health Sciences Library, McMaster University, Hamilton, ON Canada; 6 Department of Anesthesia, McMaster University, Hamilton, ON Canada; 7 Centre for Evaluation of Medicines, Hamilton, ON Canada; 8 Biostatistics Unit, St Joseph’s Healthcare-Hamilton, Hamilton, ON Canada

**Keywords:** Obesity, Type 2 diabetes, Insulin resistance, Immunometabolism, Macrophages, Skeletal muscle

## Abstract

**Objectives:**

The current global rates of obesity and type 2 diabetes are staggering. In order to implement effective management strategies, it is imperative to understand the mechanisms of obesity-induced insulin resistance and diabetes. Macrophage infiltration and inflammation of the adipose tissue in obesity is a well-established paradigm, yet the role of macrophages in muscle inflammation, insulin resistance and diabetes is not adequately studied. In this systematic review, we will examine the evidence for the presence of macrophages in skeletal muscle of obese humans and mice, and will assess the association between muscle macrophages and insulin resistance. We will identify published studies that address muscle macrophage content and phenotype, and its association with insulin resistance. We will search MEDLINE/PubMed, EMBASE, and Web of Science for eligible studies. Grey literature will be searched in ProQuest. Quality assessment will be conducted using the Systematic Review Centre for Laboratory Animal Experimentation risk of bias Tool for animal studies.

**Results:**

The findings of this systematic review will shed light on immune-metabolic crosstalk in obesity, and allow the consideration of targeted therapies to modulate muscle macrophages in the treatment and prevention of diabetes. The review will be published in a peer-reviewed journal and presented at conferences.

**Electronic supplementary material:**

The online version of this article (doi:10.1186/s13104-017-2686-6) contains supplementary material, which is available to authorized users.

## Introduction

Type 2 diabetes (T2D) is a major health concern that is driven by the obesity epidemic [1]. As population growth and longevity rates continue to advance globally, obesity-driven disorders including cardiovascular disease, stroke and T2D represent an increasing burden on individuals, societies, and healthcare systems around the world [2]. Identifying the causes of obesity-driven T2D may pave the way for targeted interventions that treat, and ideally, prevent these diseases.

The presence of obesity is known to trigger immune system activity and whole-body inflammation. This results in a low-grade, chronic inflammatory state characterized by the production of chemical attractants of immune cells called ‘chemokines’. Chemokines drive innate and adaptive immune cells to infiltrate the adipose tissue [3].

The sequence of immune cell involvement in obesity is complex. Early in the course of obesity, neutrophils enter the adipose tissue, followed by monocytes. Once monocytes sense the adipose tissue microenvironment, they differentiate to classically activated inflammatory (M1) macrophages that secrete pro-inflammatory cytokines, leading to adipose tissue inflammation and insulin resistance [3]. On the other hand, another type of macrophage, with anti-inflammatory actions, known as resident (M2) macrophage is also present in adipose tissue, and is responsible for retaining homeostasis by regulating tissue remodeling and function [4].

One theory linking the inflammatory responses in adipose tissue to muscle inflammation, and subsequent insulin resistance, suggests that there is a spillage of fatty acids and cytokines from expanding adipose tissue to the systemic vasculature. These cytokines and fatty acids are then able to elicit inflammation at distant organs including skeletal muscle and the liver [3].

Skeletal muscle plays a critical role in glucose homeostasis, and is prone to insulin resistance due to its sensitivity to lipotoxicity, glucotoxicity and inflammation. This might lead to the observed muscle insulin resistance, and eventual T2D [5].

While convincing evidence exists for the presence of macrophages and inflammation in obese adipose tissue [6], the substantiation of muscle inflammation leading to insulin resistance is less clear. Some studies have confirmed the presence of macrophages in muscle of mice [6–10] and humans [11–15], while other studies have contradicted this finding in mice [16] and humans [11, 15, 17, 18]. Even when macrophages are detected, some studies show no effect of macrophages on insulin resistance [18].

Given the uncertainty related to skeletal muscle macrophage content in obesity, a systematic review is warranted to summarize and assess the quality of the current literature that describe the muscle-immune connection in obesity and its relation with insulin resistance.

## Objectives

The objective of this systematic review is to identify and evaluate primary evidence assessing macrophage content and phenotype in skeletal muscle of obese mice and humans, in comparison to lean mice and humans.

## Methods

**Fig. 1:**
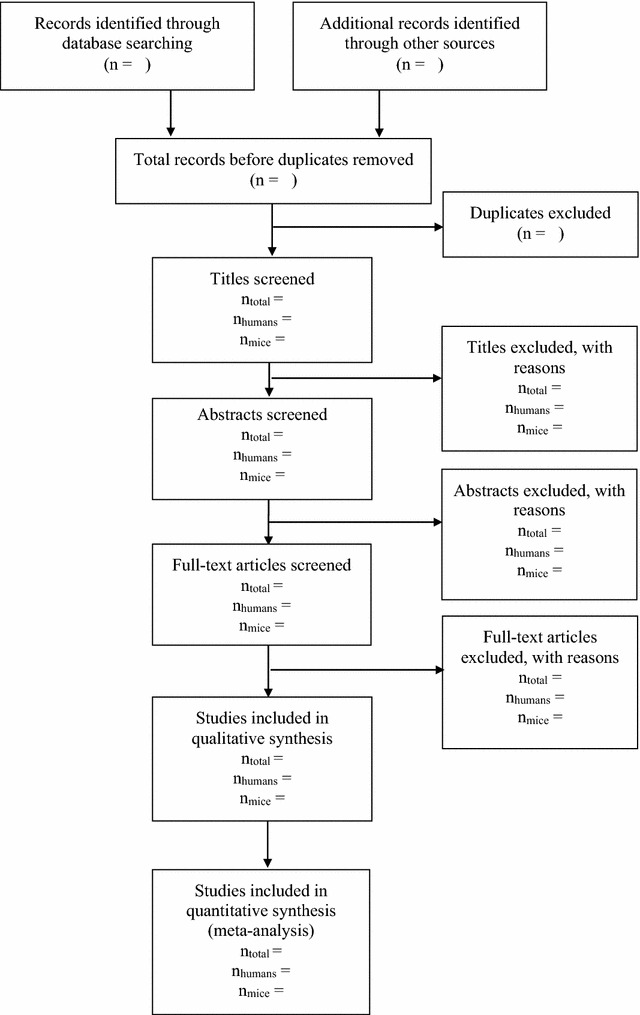
The flow diagram for the protocol

This protocol has been registered with PROSPERO (Registration Number: CRD42016033035), and is reported using guidance from the preferred reporting items for systematic review and meta-analysis protocols (PRISMA-P) [19]. The PRISMA-P checklist is available online as supplemental material (Additional file 1). The systematic review will be reported in accordance with the PRISMA guidelines [20]. Figure 1 reports the flow diagram for the protocol.

### Data sources and search strategy

An experienced Health Sciences Librarian (LB) will be consulted in developing the search strategy. We will perform electronic data searches using adapted medical subject headings and key terms for each database, as per the comprehensive search strategy (Table 1). We will search MEDLINE/PubMed, EMBASE, and Web of Science to identify eligible studies for the review. The ProQuest database will be searched as a source of grey literature. We will review the reference lists of all included studies, to ensure that we are covering all relevant literature. We will also search for additional publications by authors of eligible studies to identify relevant primary research.

**Table 1:** Search strategy for retrieval of references from MEDLINE

Database	Search strategies
MEDLINE	1. Muscles/
	2. muscle*.ti.ab.kf.
	3. exp Muscle, Skeletal/
	4. (abdominal muscle* or pelvic floor or rectus abdominis or back muscles or intermediate back muscle* or paraspinal muscle* or superficial back muscle* or deltoid muscle* or facial muscle* or laryngeal muscle* or masticatory muscle* or masseter muscle* or pterygoid muscle* or temporal muscle* or neck muscle* or oculomotor muscle* or palatal muscle* or pectoralis muscle* or pharyngeal muscle* or upper esophageal sphincter* or velopharyngeal sphincter* or psoas muscle* or quadriceps muscle* or respiratory muscle* or diaphragm* or intercostal muscle* or rotator cuff* or stapedius or tensor tympani).ti.ab.kf.
	5. or/1-4
	6. Macrophages/
	7. Epithelioid Cells/
	8. Foam Cells/
	9. Giant Cells, Foreign-Body/
	10. Giant Cells, Langhans/
	11. macrophage*.ti.ab.kf.
	12. (epithelioid cell* or foam cell* or (giant cell* adj3 foreign-body) or (giant cell* adj3 langhans)).ti.ab.kf.
	13. or/6-12
	14. exp Obesity/
	15. obese.ti.ab.kf.
	16. obesity.ti.ab.kf.
	17. overweight.ti.ab.kf.
	18. over weight.ti.ab.kf.
	19. Overweight/
	20. Metabolic Syndrome X/
	21. metabolic syndrome x.mp.
	22. or/14-21
	23. 5 and 13 and 22
	24. remove duplicates from 23

### Eligibility criteria for studies

This review will include studies with case–control, prospective cohort, retrospective cohort, and cross-sectional designs. There will be no restrictions on language of publication. We will include pilot and feasibility studies, conference abstracts and posters if relevant to the review question by contacting the authors and requesting the data. We will exclude case reports. We will include all studies that examine muscle macrophage content and phenotype in lean and overweight/obese humans and mice. Studies with human participants of all ages, sexes and ethnicities will be included. We will exclude studies that report on patients who have hypertension, chronic renal disease, autoimmune disease, cancer, pregnancy, smokers, and athletes. Recipients of parenteral nutrition, steroids, anti-inflammatory or immunomodulating therapies will also be excluded.

For estimation of muscle macrophage content and phenotype in humans and mice, studies will be eligible if using measurements such as quantitative or semi-quantitative real-time polymerase chain reaction, flow cytometry, western blot, and immunohistochemistry. Human studies will be deemed eligible if total obesity is measured using one of the following measures: body mass index (BMI), total fat mass measured by bioelectrical impedance, or dual x-ray absorptiometry (DXA) scans. Furthermore, human studies will be included if measuring regional adiposity by waist-to-hip ratio, DXA, computerized tomography (CT) or magnetic resonance imaging (MRI) estimations of visceral fat mass. In mice, studies determining weight or adiposity using DXA, CT or MRI scans will be eligible for this review.

Eligible assessments of insulin resistance will include direct measures or surrogate measures. Direct measures such as hyperinsulinemic euglycemic clamp or glucose challenge using minimal model will be considered. Eligible surrogate measures include the following: fasting insulin, homeostasis model index of insulin resistance (HOMA-IR), the quantitative insulin check index of insulin sensitivity (QUICKI), oral glucose tolerance test-derived measures, frequently sampled intravenous glucose tolerance test, Matsuda, Stumvoll, Belfiore, and Avignon indices [21]. Surrogate markers of insulin resistance will only be considered in population- or clinical-based cross-sectional studies, as they are not reliable in longitudinal metabolic studies [22]. If a study involves an intervention, where muscle samples were obtained before and after the intervention, we will use the data from the pre-intervention samples.

### Outcome measures

We will assess the following primary and secondary endpoints of interest:

#### Primary

The primary outcome measures include (1) macrophage content in skeletal muscle during obesity in humans and mice and (2) the association between muscle macrophage content and insulin resistance in obese mice and humans. For macrophage markers, we will include cluster of differentiation 68 (CD68) in humans and Adhesion G Protein-Coupled Receptor E1 (Emr1, also known as F4/80) in mice. CD68 is a transmembrane glycoprotein that is selectively expressed in macrophages, and has been widely used in macrophage identification [11]. Emr1 is a widely used marker of murine macrophages [23].

#### Secondary

We will assess the phenotype of macrophages as they present in skeletal muscle during obesity, using a different set of markers. For M1 macrophages, we will use Integrin, Alpha X (Complement Component 3 Receptor 4 subunit) (CD11c), a molecule that is important in phagocytosis and adhesion of macrophages, as marker of inflammatory macrophages [9, 24]. In humans, M2 macrophages will be detected using the multifunctional enzyme transglutaminase 2 (TGM2) which, when combined with mannose receptor C type 1 (MRC1), CD206 and CD68, can identify human M2 macrophages with standard Immunohistochemical double staining techniques [25]. M2 macrophage markers in mice include TGM2, resistin-like molecule-alpha (FIZZ1) [26], arginase-1 (Arg-1), and chitinase-3-like protein-3 (Chi3l3, also known as Ym1) with the latter having no human homologs [27].

If studies identify other markers of macrophage phenotype, they will be included.

### Data management

We will conduct title and abstract screening, full-text review and data abstraction using Microsoft Excel. The authors will develop and pilot test the data abstraction forms on Excel to ensure validity.

### Study selection

Two reviewers will independently complete title and abstract screening to identify relevant articles based on the eligibility criteria. Articles deemed eligible during title and abstract screening will be subject to full-text review. Reviewers will resolve disagreements during the study selection process through discussion to consensus. A third reviewer will be consulted if no resolution is reached. We will contact authors of relevant studies if sufficient data are not available to assess eligibility based on the published work. Articles that do not meet eligibility criteria will be excluded from the review, and reasons for exclusion will be documented and reported in a flow diagram, as per PRISMA guidelines [20]. We will calculate a kappa statistic to determine inter-rater agreement for each stage of screening to demonstrate the level of agreement between reviewers.

### Data extraction

Two reviewers will extract data independently from included studies using a predetermined and pilot-tested data extraction form. The following data will be extracted from all studies: study authors, journal name, year of publication, funding source, country, study design, number of participants/mice in obese and lean groups, age, sex, muscle type subject to biopsy, and measures of weight and adiposity. We will also extract data about the macrophage markers used to determine macrophage content in muscle and the techniques used to measure the markers. Additionally for human studies, we will extract the type of participants (e.g. clinic, school, community), ethnicity, and fitness level. For mouse studies, we will extract the exact genetic background of the mice, nature of genetic alteration (if applicable), feeding regimens, water regimens and access, activity and metabolic monitoring, and housing conditions and light/dark cycles.

For both human and mouse studies, we will also document the statistical methods used for data analyses, and adjustments made for confounders, and authors’ conclusions based on results. If the study is a trial with pre- and post-intervention phases, we will only include data from the pre-intervention phase. Measures of association for macrophage content and insulin resistance will be reported as provided by the authors.

### Quality assessment

We will assess quality using the Systematic Review Centre for Laboratory Animal Experimentation (SYRCLE) risk of bias tool adapted for non-intervention animal studies [28]. SYRCLE’s risk of bias tool assigns *high, unclear* or *low* risk of bias to studies for factors such as sequence generation, allocation concealment, random housing, blinding, incomplete outcome data, selective outcome reporting and other sources of bias.

For human participants, the Newcastle–Ottawa Scale will be used to assess quality of included studies [29, 30]. This scale is based on the incorporation of three paradigms, including the selection of groups studied, comparability of these groups, ascertainment of exposure in case–control studies, and outcomes for cohort studies. Its scores are based on a star system, with top-ranking studies receiving nine stars.

### Data synthesis and assessment of heterogeneity

Findings of included studies will be reported as a narrative summary and we will provide quantitative summary using meta-analysis, if possible. Studies will be combined to determine the association between macrophage content and insulin resistance based on similarities in design, methods, exposure and outcome measurements. We will use a random effects model, which accounts for both within-study and between-study variability, as we expect heterogeneity in the literature [31]. We will use RevMan 5.3 software to generate forest plots to represent data graphically if applicable. Continuous outcomes will be calculated using standardized mean difference with standard deviation. Dichotomous outcomes will be pooled using odds ratio with 95% confidence interval.

Heterogeneity will be assessed using the I^2^ statistic and a cutoff value of >40% will be considered substantial heterogeneity, as per the Cochrane Handbook [32]. If studies have high selective reporting bias, a sensitivity analysis will be performed by removing studies with high risk of bias to assess the impact on meta-analysis results [33].

Egger’s test will be used to assess for publication bias, and funnel plots will be generated if there are 10 or more studies reporting the outcome of interest [34].

As muscle is a critical organ for whole-body insulin action, understanding macrophage existence in skeletal muscle, their phenotype, and their role in muscle insulin resistance will make macrophages a potential therapeutic target to treat and prevent obesity and diabetes.

## Limitations

- If the studies have high heterogeneity, it may be difficult to combine data and perform meta-analysis.
- If sample sizes are small, this will limit the quality of the conclusions drawn from this review.

## Additional file

**Additional file 1.** PRISMA-P (Preferred Reporting Items for Systematic review and Meta-Analysis Protocols) 2015 checklist: recommended items to address in a systematic review protocol.
